# Identifying youth at high risk for sexually transmitted infections in community-based settings using a risk prediction tool: a validation study

**DOI:** 10.1186/s12879-021-06937-4

**Published:** 2021-12-08

**Authors:** Katharina Kranzer, Victoria Simms, Ethel Dauya, Ioana D. Olaru, Chido Dziva Chikwari, Kevin Martin, Nicol Redzo, Tsitsi Bandason, Mandikudza Tembo, Suzanna C. Francis, Helen A. Weiss, Richard J. Hayes, Constancia Mavodza, Tsitsi Apollo, Gertrude Ncube, Anna Machiha, Rashida Abbas Ferrand

**Affiliations:** 1 Clinical Research Department, Faculty of Infectious and Tropical Diseases, London School of Hygiene and Tropical Medicine, London, UK; 2 Biomedical Research and Training Institute, Harare, Zimbabwe; 3 Division of Infectious and Tropical Medicine, Medical Centre of the University of Munich, Munich, Germany; 4 MRC International Statistics and Epidemiology Group, Department of Infectious Disease Epidemiology, London School of Hygiene and Tropical Medicine, London, UK; 5 Department of Global Health and Infection, Brighton and Sussex Medical School, Brighton, UK; 6 Department of Public Health and Policy, London School of Hygiene and Tropical Medicine, London, UK; 7 AIDS and TB Unit, Ministry of Health and Child Care, Harare, Zimbabwe

**Keywords:** Sexually transmitted infections, Adolescents, Screening, Risk prediction tool

## Abstract

**Background:**

*Chlamydia trachomatis* (CT) and *Neisseria gonorrhoeae* (NG) are the most common bacterial sexually transmitted infections (STIs) worldwide. In the absence of affordable point-of-care STI tests, WHO recommends STI testing based on risk factors. This study aimed to develop a prediction tool with a sensitivity of > 90% and efficiency (defined as the percentage of individuals that are eligible for diagnostic testing) of < 60%.

**Methods:**

This study offered CT/NG testing as part of a cluster-randomised trial of community-based delivery of sexual and reproductive health services to youth aged 16–24 years in Zimbabwe. All individuals accepting STI testing completed an STI risk factor questionnaire. The outcome was positivity for either CT or NG. Backwards-stepwise logistic regression was performed with p ≥ 0.05 as criteria for exclusion. Coefficients of variables included in the final multivariable model were multiplied by 10 to generate weights for a STI risk prediction tool. A maximum likelihood Receiver Operating Characteristics (ROC) model was fitted, with the continuous variable score divided into 15 categories of equal size. Sensitivity, efficiency and number needed to screen were calculated for different cut-points.

**Results:**

From 3 December 2019 to 5 February 2020, 1007 individuals opted for STI testing, of whom 1003 (99.6%) completed the questionnaire. CT/NG prevalence was 17.5% (95% CI 15.1, 19.8) (n = 175). CT/NG positivity was independently associated with being female, number of lifetime sexual partners, relationship status, HIV status, self-assessed STI risk and past or current pregnancy. The STI risk prediction score including those variables ranged from 2 to 46 with an area under the ROC curve of 0.72 (95% CI 0.68, 0.76). Two cut-points were chosen: (i) 23 for optimised sensitivity (75.9%) and specificity (59.3%) and (ii) 19 to maximise sensitivity (82.4%) while keeping efficiency at < 60% (59.4%).

**Conclusions:**

The high prevalence of STIs among youth, even in those with no or one reported risk factor, may preclude the use of risk prediction tools for selective STI testing. At a cut-point of 19 one in six young people with STIs would be missed.

## Introduction

*Chlamydia trachomatis* and *Neisseria gonorrhoeae* are the most common bacterial sexually transmitted infections (STIs) worldwide. These conditions have important sexual, reproductive, and maternal-child health consequences, including genital symptoms, pregnancy complications, infertility, enhanced HIV transmission, and psychological effects [1–8]. Importantly both chlamydia and gonorrhoea are curable with well-tolerated short-course antibiotics.

In 2016, there were an estimated 127.2 million (95% uncertainty interval (UI): 95.1–165.9 million) chlamydia and 86.9 million (95% UI: 58.6–123.4 million) gonorrhoea infections globally, with prevalence varying by World Health Organization (WHO) region [9]. Chlamydia prevalence estimates among 15–49 year olds in the African region are the second highest globally at 5.0% (95% UI: 3.8–6.6) in women and 4.0% (95% UI: 2.4–6.1) in men. The African region is estimated to have the highest prevalence of gonorrhoea globally with a prevalence of 1.9% (95% UI: 1.3–2.7) in women and 1.6% (95% UI: 0.9–2.6) in men. Systematic reviews focusing on women in sub-Saharan Africa revealed a prevalence of chlamydia of 3.3–7.8% and gonorrhoea of 2.2–4.2% [10, 11]. However, comparable data for men are scarce. Notably, youth are at much higher risk of STIs than adults [9, 12–14]. An individual participant data meta-analysis including women participating in 18 HIV prevention studies in sub-Saharan Africa recruited mostly before 2010 showed that chlamydia and gonorrhoea prevalence was generally higher among the 15-24 year olds compared to the 25–49 year olds [15].

The WHO Global Health Sector Strategy on STIs 2016–2021 (Global Strategy), provides goals, targets, and priority actions for curtailing the STI epidemic [16]. A priority action for countries is the implementation and scale-up of services aimed at early diagnosis of STIs to ensure effective medical treatment and prevent further transmission. Early diagnosis of STIs is challenging, given that most STIs are asymptomatic especially in women [17–19]. In the absence of affordable point-of-care tests for STIs, universal screening remains rare in resource-constrained settings. An approach promoted by WHO is to offer STI testing to asymptomatic individuals based upon risk factors or risk prediction tools [16].

Clinical prediction rules for STIs have been successfully developed for high-income settings to allow for a so-called “selective screening” approach [20, 21]. This approach is aimed at minimising costs associated with testing low-risk individuals while detecting most infections. Thresholds of 60% efficiency (defined as percentage of individuals that are eligible for diagnostic testing based on predictive criteria) and 90% sensitivity have been proposed as ideal benchmarks for clinical prediction tools in the context of STIs [20, 22, 23].

Previous STI risk prediction tools administered by healthcare providers in Africa have been developed using an ad-hoc approach and were found to have a poor sensitivity; none have been developed specifically for youth [19, 24]. We aimed to develop a clinical prediction tool for STIs specifically targeting youth in Zimbabwe.

## Methods

### Study design and setting

This study was nested within a cluster-randomised trial (CHIEDZA) of an integrated package of HIV and sexual and reproductive health (SRH) services for youth delivered in community-based settings in Zimbabwe (registered in clinical trials.gov: NCT03719521). Individuals aged 16–24 years living within an intervention cluster are eligible to receive an integrated package of SRH services including HIV testing, HIV treatment and adherence support, contraception, pregnancy testing, syndromic management of STIs, menstrual health information and products, condoms and general health counselling. Individuals older than 24 years of age at a repeat visit, who were less than 25 years at the first visit are also eligible to accessing the services. Testing for gonorrhoea and chlamydia was offered to all clients accessing CHIEDZA services over a limited period of time. Treatment of STIs and HIV is provided according to national guidelines. All services are offered free of charge.

The trial is being conducted in three provinces (Harare, Mashonaland East and Bulawayo), with each province containing eight geographically demarcated clusters randomised 1:1 to four intervention and four standard of care (routine, existing services) clusters. The intervention is delivered once weekly (on the same day each week) at a community centre in each intervention cluster by a team of nurses, community health workers, youth workers and a counsellor.

This sub-study assessing STI risk factors was conducted in eight intervention clusters in Harare and Mashonaland East.

### STI testing

All individuals accessing CHIEDZA services were non-selectively offered testing for gonorrhoea and chlamydia if they had not tested within the 6 months prior to the visit, regardless of whether they had symptoms or risk factors for STIs. Those who accepted testing were asked to provide a urine sample which was tested using the GeneXpert platform (Cepheid, Sunnyvale, CA, USA) [9]. All individuals were given the option to pick up their result the following week, and individuals with a positive result were actively contacted by phone and asked to visit the centre. Positive test results were not disclosed over the phone, but only provided face to face. Partner notification (PN) slips were given to those who had a positive STI test result and all partners were offered treatment. Individuals who reported STI symptoms were treated according to national guidelines for syndromic management but were also offered testing for gonorrhoea and chlamydia [25].

### Risk factors for STIs

All individuals accepting STI testing were approached by the study team and asked if they would like to participate in the risk factor sub-study. Those consenting were asked to answer a short questionnaire (11 items) regarding current relationship status, number of sexual partners, concurrent partners, condom use, use of contraception, previous or current pregnancies and STI risk perception. HIV status was obtained from the CHIEDZA dataset. The questions in the questionnaire were informed by studies developing risk prediction tools for chlamydia and gonorrhoea infection in high-income settings [20, 21] and studies investigating risk factors for those infections in sub-Saharan Africa [13, 17, 19, 24, 26, 27]. The questionnaire was administered by a research assistant not involved in STI testing or delivering other CHIEDZA services.

### Data analysis

The outcome was positivity for either *C. trachomatis* or *N. gonorrhoeae*, combined as one variable. For variables that only applied to women (past pregnancy and use of hormonal contraception) males were coded ‘no’ for multivariable analysis. Univariable logistic regression was used to estimate the odds ratios (OR) and 95% confidence intervals (95% CI) for the association between STI infection and each of the 11 risk factors determined by the questionnaire and HIV status, age and sex. Variables that were associated with the outcome at 10% significance level in the univariable analysis were included in a multivariable model and then removed sequentially using backwards stepwise logistic regression until all remaining variables were associated with the outcome at < 5% significance. For ordinal variables p-values were calculated with a Wald test. Coefficients of variables included in the final multivariable model were multiplied by 10 to generate weights, and the weights were added for each individual to create an STI risk score. A maximum likelihood Receiver Operating Characteristic (ROC) model was fitted, with the continuous score variable divided into 15 categories of equal size. All possible cut-points of the risk prediction tool were evaluated for sensitivity, specificity, efficiency (proportion of the sample who screen positive) and number needed to test to obtain one positive result.

From a pilot study, the prevalence of the outcome was estimated at 17% [28]. A sample size of 1000 gave 80% power to detect a risk ratio of 1.7 for a risk factor with 10% prevalence, and 80% power to detect a risk ratio of 2.0 for a risk factor with 5% prevalence.

## Results

Between 3 December 2019 and 5 February 2020; 1007 individuals opted for STI testing of whom 1003 gave consent for data on risk factors to be collected. The majority of these were female (78.7%, n = 789) and aged 20–24 (58.2%, n = 584) years, similar to the demographic profile of those accessing CHIEDZA services. HIV status was known for 957 participants, among whom HIV prevalence was 5.2% (n = 50). Of these, 36 (72.0%) were previously diagnosed and taking antiretroviral therapy (ART), and 14 (28.0%) were newly diagnosed through CHIEDZA.

Prevalence of *C. trachomatis* and/or *N. gonorrhoeae* infection was 17.5% (95% CI 15.1–19.8) (n = 175), of whom 14.8% (95% CI 12.6–17.1) (n = 148) tested positive for *C. trachomatis* and 4.1% (n = 41) (95% CI 2.9–5.5) for *N. gonorrhoeae*, with 1.4% (n = 14) testing positive for both infections. In total 39 of the 1003 participants (3.8%) reported STI symptoms and 21 received syndromic management, of whom 13 subsequently tested positive for either *C. trachomatis*, *N. gonorrhoeae* or both.

In univariable analysis, older age, being female, being in a relationship or widowed/divorced, number of lifetime sexual partners and number of sexual partners in the past three months, having had a new sexual partner in the past three months, history of STI treatment, history of STI treatment of the partner, occasional condom use, perceived high STI risk, positive HIV status and past or current pregnancy were all associated with having an STI (p < 0.1; Table 1). The multivariable analysis including all these variables showed an independent significant association between STI infection and being female, relationship status, number of lifetime sexual partners, HIV status, perceived STI risk and past or current pregnancy. These variables were included in the final multivariable model. Odds ratios for associations between risk factors and having an STI ranged between 1.23 and 3.98 (Table 1).

**Fig. 1:**
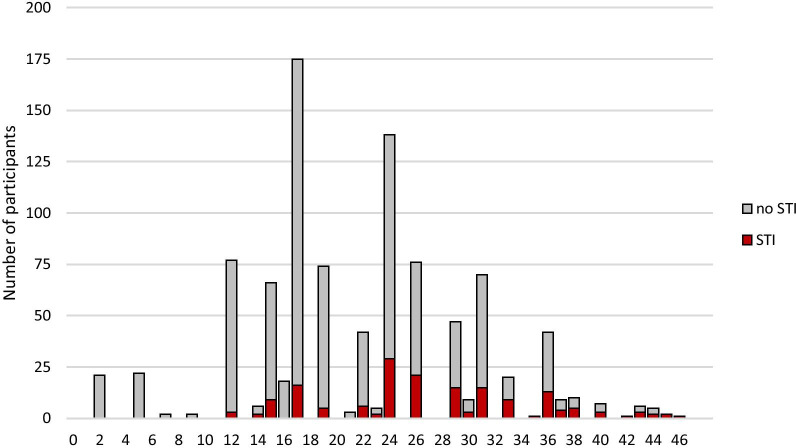
Distribution of STI risk scores. Red bars: tested positive for STI, grey bar: tested negative for STI, red line: efficiency (proportion scoring at or above the cut-point), grey line: sensitivity, blue line specificity, black dotted line: cut-point of 23

The STI risk prediction tool generated from the final model is shown in Table 2. The score ranged from 2 for single participants with no other risk factors to 46 for those with all six risk factors (Fig. 1). For example, an HIV negative woman with a boyfriend who had 1 lifetime sexual partner, perceived herself at low risk and had never been pregnant would score 10 + 5 + 7 = 22. The maximum possible score for males was 41. A score of 0 would only be possible for a married male with no lifetime sexual partners, which did not occur in the dataset.

**Table 1:** Logistic regression models for association of risk factors with STI prevalence

Variable		N with STI/ total N (STI prevalence)	Univariable		Multivariable 1 (N = 957)		Multivariable 2 (N = 957)	
			OR (95% CI)	p	OR	p	OR	p
Age group	16–19	61/419 (14.6)	1	0.042	1	0.95		
	20–25	114/584 (19.5)	1.42 (1.01, 2.00)		1.01 (0.68, 1.51)			
Sex	Male	23/214 (10.8)	1	0.004	1	0.001	1	0.001
	Female	152/789 (19.3)	1.98 (1.24, 3.16)		2.77 (1.54, 4.98)		2.64 (1.52, 4.57)	
Relationship status	Single	21/177 (11.9)	0.65 (0.39, 1.10)	0.005	1.48 (0.72, 3.04)	0.02	1.23 (0.67–2.27)	0.007
	Boyfriend/girlfriend	70/368 (19.0)	1.14 (0.80, 1.64)		1.82 (1.05, 3.12)		1.70 (1.09–2.63)	
	Married	74/434 (17.1)	1		1		1	
	Divorced/widowed	10/24 (41.7)	3.47 (1.49, 8.12)		4.10 (1.50, 11.25)		3.71 (1.51–9.06)	
New partner in the last 3 months	Yes	35/157 (22.3)	1.45 (0.95, 2.20)	0.083	0.69 (0.36, 1.30)	0.25		
	No	129/846 (16.6)	1		1			
No of sexual partners in last 3 months	0	31/293 (10.6)	1	< 0.001	1	0.16		
	1	119/623 (19.1)	2.00 (1.31, 3.04)		1.07 (0.54, 2.12)			
	2+	25/87 (28.7)	3.41 (1.88, 6.18)		2.09 (0.83, 5.25)			
No of lifetime partners	0	11/186 (5.9)	1		1	0.004	1	
	1	54/388 (13.9)	2.57 (1.31, 5.04)	< 0.001	2.19 (0.85, 5.64)		2.09 (0.98, 4.44)	< 0.001
	2 +	110/429 (25.6)	5.49 (2.87, 10.47)		3.89 (1.47, 10.29)		3.98 (1.93, 8.19)	
History of STI treatment	Yes	22/79 (27.9)	1.94 (1.15, 3.28)	0.012	0.75 (0.40, 1.40)	0.36		
	No	153/924 (16.6)	1		1			
Partner ever had an STI	Yes	17/43 (39.5)	3.32 (1.76,6.26)	< 0.001	1.90 (0.88, 4.07)	0.10		
	No	158/960 (16.5)	1		1			
HIV status (N = 957)	Positive	18/50 (36.0)	2.79 (1.53, 5.11)	< 0.001	2.05 (1.05, 4.01)	0.04	1.95 (1.02, 3.72)	0.04
	Negative	152/907 (16.8)	1		1		1	
Use of condoms (N = 817)	Always	15/116 (12.9)	0.70 (0.38, 1.28)	0.004	0.65 (0.32, 1.35)	0.12		
	Sometimes	82/319 (25.7)	1.63 (1.13, 2.34)		1.27 (0.81, 1.99)			
	Never	67/382 (17.5)	1		1			
Perceived STI risk	None/low	93/721 (12.9)	1	< 0.001	1	0.01	1	< 0.001
	Medium/high	82/282 (29.1)	2.77 (1.98, 3.88)		1.67 (1.12, 2.49)		1.98 (1.37, 2.87)	
Use of hormonal contraception (N = 789)	Yes	87/451 (19.3)	1.00 (0.70, 1.44)	0.98				
	No	65/338 (19.2)	1					
Past or current pregnancy (N = 789)	Yes	13/36 (36.1)	2.50 (1.23, 5.05)	0.011	2.70 (1.27, 5.78)	0.01	2.55 (1.21, 5.39)	0.014
	No	139/753 (18.5)	1		1		1	

Multivariable Model 1: includes all potential risk factors and Multivariable Model 2: includes potential risk factors that were associated with STI infection in Model 1 ( female gender, relationship status, number of lifetime sexual partners, HIV status, perceived STI risk and past/current pregnancy)

**Table 2:** STI risk score variable weightings for variables with p < 0.10 in the final multivariable logistic regression model

		β-Coefficient	Weighting
Sex	Female	0.97	10
Relationship status (vs. married)	Single	0.21	2
	Boyfriend/girlfriend	0.53	5
	Divorced/widowed	1.31	13
Number of lifetime partners (vs. 0)	1	0.74	7
	≥ 2	1.38	14
HIV status	Positive	0.67	7
Perceived risk of STI	Medium/high	0.68	7
Past pregnancy	Yes	0.94	9

**Fig. 2:**
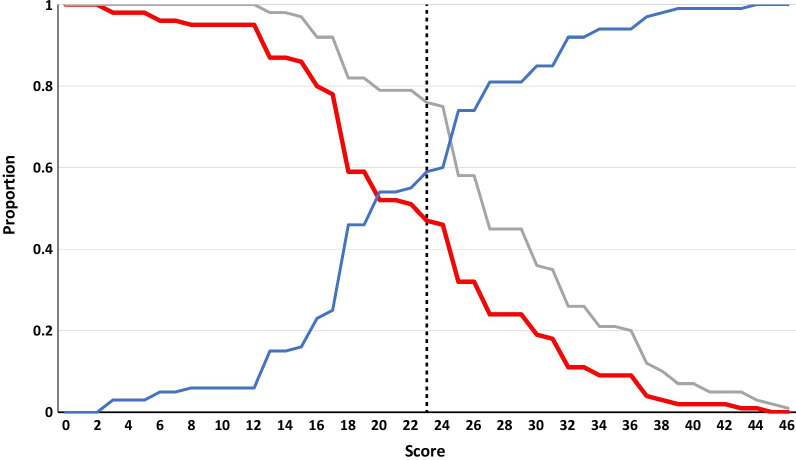
Sensitivity and specificity of all risk score cut-points. Red line: efficiency (proportion scoring at or above the cut-point), grey line: sensitivity, blue line specificity, black dotted line: cut-point of 23

**Fig. 3:**
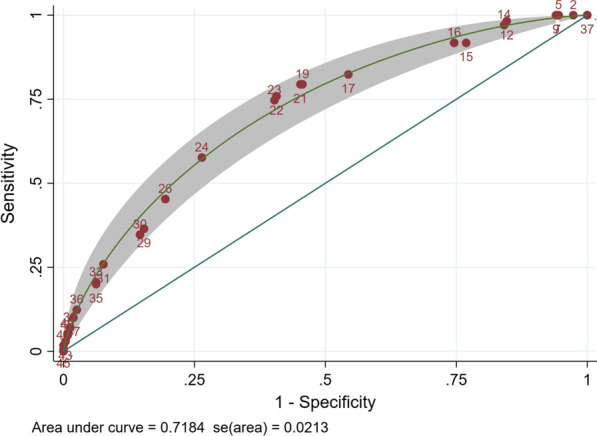
ROC plot of STI risk score

The sensitivity, specificity and efficiency of all possible cut-points is shown in Fig. 2, and the ROC curve with 95% CI is shown in Fig. 3. The area under the curve is 0.72. Two cut-points for the risk tool were chosen: 23 for optimised sensitivity (75.9%) and specificity (59.3%), and 19 to meet the benchmark of maximising sensitivity (82.4%) while keeping efficiency (59.4%) at less than 60% (Table 3). The number needed to screen to diagnose one STI was 3.5 for a cut-point of 23 and 4.1 for a cut-point of 19.

**Table 3:** Performance of STI risk score at different cut-points

	Cutpoint 19		Cutpoint 23	
	% (n/N)	95% CI	% (n/N)	95% CI
Sensitivity	82.4% (140/170)	75.8, 87.8	75.9% (129/170)	68.7, 82.1
Specificity	45.6% (359/787)	42.1, 49.2	59.3% (467/787)	55.8, 62.8
Yield	24.7% (140/568)	21.2, 28.4	28.7% (129/449)	24.6, 33.2
Efficiency	59.4% (568/957)	56.2, 62.5	46.9% (449/957)	43.7, 50.1
NNT	4.1		3.5	

Efficiency = proportion of population that is tested

NNT = number needed to test to obtain one positive result

## Discussion

We found a high prevalence of *C. trachomatis* and/or *N. gonorrhoeae* infection among young people attending a community based SRH service in urban and peri-urban Zimbabwe. Notably, only 2.1% (21/1003) of participants were found to be positive for an STI syndrome and treated accordingly. The prevalence of *C. trachomatis* infection was almost three times higher than the WHO estimates for the African region, but comparable with studies conducted among young women in South Africa [9, 26, 29]. The prevalence of *N. gonorrhoeae* was similar to the recent estimates from the Spectrum-STI model for Zimbabwe of 3.8% (95% CI 1.8–6.7%) [30].

With non-selective testing the number needed to be tested to diagnose an STI was 5.7. This decreased to 4.1 using an STI risk prediction tool cut-point of 19 and 3.5 for a risk prediction tool cut-point of 23. While the STI risk prediction tool with a cut-point of 19 had the desired efficiency of < 60%, the sensitivity was suboptimal at 82%. This is because the prevalence of STIs even among clients with the lowest possible STI risk in this population was relatively high. For example STI prevalence was 5.9% among those who reported that they had never had sex and 13.9% among those who only had one lifetime sexual partner. The high STI prevalence among young people without reported risk factors in this population suggests that non-selective testing may be more appropriate than applying a risk prediction tool.

Many recently-published studies show high prevalence of STIs in general African populations but none has developed a risk prediction tool [13, 17, 26, 27, 29, 31]. To our knowledge, this is the first study globally that has attempted to develop a risk prediction tool in youth, who are a high-risk group for these STIs. A recent Kenyan study among men who have sex with men developed a risk tool for anorectal *C. trachomatis* and/or *N. gonorrhoeae* infection reaching a sensitivity of 86% at an efficiency of 61% [32]. The proposed risk tool came close to the ideal benchmarks for clinical prediction rule performance for STIs of > 90% sensitivity and < 60% efficiency [20, 22, 23]. The only study examining the performance of STI risk prediction tools among African women was conducted in 1994 in Tanzania and reported sensitivities between 10 and 29% which are inadequate [24].

A recent study enrolling Rwandan women in 2016–2017 compared the performance of a new diagnostic algorithm (‘WISH’ algorithm) with reference standard diagnostic testing [19]. The WISH algorithm was predefined at the start of the study; women were considered positive according to the algorithm if they met one or more of the following criteria: currently pregnant, exchanged sex for money or goods in the past 12 months, new sexual partner in the past 3 months, or vaginal discharge with an offensive smell or pelvic inflammatory disease observed by a physician. The prevalence of *C. trachomatis* and/or *N. gonorrhoeae* was 14% using vaginal swabs investigated by GeneXpert and the sensitivity and efficiency of the WISH algorithm was 75% and 56% respectively. While the population in the WISH study is not truly comparable to our study, low sensitivities of both the WISH algorithm and our risk prediction tool would result in missing one in four individuals with STIs using the WISH algorithm and one in six using our risk prediction tool.

A previous study conducted in the same population in Zimbabwe found that only 0.5% of youth were treated for a STI syndrome and less than 5% reported symptoms when asked specifically [28]. Given the low prevalence of reported symptoms, questions about symptoms were not included in the questionnaire. However the questionnaire used in this study included a broad range of possible risk factors and was informed by studies investigating risk factors for STIs in Africa and STI risk prediction tools developed for high income settings [13, 17, 19–21, 24, 26, 27]. Questions about sexual behaviour are often subject to social desirability bias resulting in under- or mis-reporting [33–35]. This may limit the sensitivity of risk prediction tools based on questions about sexual behaviour. While computer-assisted survey instruments rather than self- or interviewer-administered questionnaires may reduce social desirability bias, these may be more difficult to implement in low-resource settings [33–35].

Unsurprisingly STI risk factors in this study were closely associated with each other and with the presence of *C. trachomatis* and/or *N. gonorrhoeae* infection in univariable analysis. Due to the co-linearity of risk factors only six independent risk factors were included in the multivariable analysis. The model that was developed was applicable to both sexes even though one risk factor, pregnancy, only applied to women. A sensitivity analysis restricted to women resulted in the same five risk factors for predicting STIs, which is reassuring regarding the robustness of the model. However, we could not conduct a sensitivity analysis for men only given the limited number of men in the study (n = 214). This is a limitation of our study, but reflects the reality that men are less likely to access services [36, 37].

The strengths of this study include the large sample size and high participation rate among those opting for STI testing. Also the study was embedded within a population-based SRH service offered in eight communities in two provinces accessed by youth without pre-selection on the basis of STI risk, which makes the findings generalisable to similar settings. We focused on two highly prevalent STIs that cause significant morbidity and mortality. However, we did not include *Trichomonas vaginalis*, which is highly prevalent especially in women in sub-Saharan Africa [13, 26, 38]. Also for logistic reasons we used urine instead of vaginal swabs. This may have resulted in some STIs being missed.

Importantly we decided against a test and train approach, which is usually used when developing risk prediction tools [39]. This is because our analysis demonstrated that even the most optimal risk prediction tool with a cut-point of 19 was unacceptable.

In view of the high prevalence of STIs among youth in sub-Saharan Africa, non-selective diagnostic testing as opposed to selective testing following the application of a risk prediction tool seems the most promising and appropriate approach. This approach will require true point-of-care STI diagnostics, which are currently available only for *Trichomonas vaginalis* and syphilis [40, 41]. While the GeneXpert platform for *C. trachomatis* and *N. gonorrhoeae* testing does not require expert skills, is easy to use and widely available in sub-Saharan Africa, the time to result (90 min) is prohibitive for the test to be used as a true point-of-care test [42]. However, other molecular tests for chlamydia and gonorrhoea with rapid time to results (30 min) have been successfully trialled in high-income settings [43]. Universal point-of-care STI testing and immediate single dose treatment for those testing positive should be considered as a strategy for curbing the STI epidemic in youth.

## Data Availability

Individual, anonymised participant data and a data dictionary will be available through The London School of Hygiene and Tropical Medicine repository (Data Compass) 12 months after publication of results. The datasets used and/or analysed during the current study available from the corresponding author on reasonable request.
